# Nursing students’ academic burnout: its prevalence and association with self-efficacy, psychological distress, and quality of learning experience

**DOI:** 10.1186/s12912-025-03242-2

**Published:** 2025-06-03

**Authors:** Shaherah Yousef Andargeery, Elaf Abdulrahman Altaweel, Fatima Salem Alkorbi, Lamia Abdulaziz Alyousef, Bushra Fayez Alanazi, Sara Haroon Idriss, Sara Salem Alqahtani, Lana Mohammed Alkhamis

**Affiliations:** 1 Department of Nursing Management and Education, College of Nursing, Princess Nourah bint Abdulrahman University, P.O. Box 84428, Riyadh, 11671 Saudi Arabia; 2 College of Nursing, Princess Nourah bint Abdulrahman University, P.O. Box 84428, Riyadh, 11671 Saudi Arabia

**Keywords:** Student burnout, Psychological distress, Self-efficacy, Learning health system, Psychological burnout

## Abstract

**Introduction:**

Academic burnout is a prevalent issue affecting students across different disciplines, including nursing students. Academic burnout negatively impacts students’ clinical performance, patient satisfaction, and care quality.

**Aim:**

This study aimed at examining the differences in the mean scores of students’ academic burnout on their demographic characteristics, and evaluating the relationship between academic burnout, self-efficacy, psychological distress, and quality of learning experience among undergraduate nursing students.

**Methodology:**

A cross-sectional design with a purposive sampling technique was used to conduct the study. The questionnaire was sent through Microsoft Forms including demographic questions, Maslach-Burnout Inventory- Student Survey, General Self-Efficacy Survey, Kessler Psychological Distress Scale, and Quality of Learning Experience Scale to measure the variables.

**Results:**

The total number of participants was 286 nursing students. The results depicted statistically significant effects of three different English proficiency levels on academic burnout F(2, 283) = 4.328, *p* =.014), program of study (t(284) = 2.270, *p* =.024, 91% CI: 0.55 to 10.45), GPA (t(284) = 2.011, *p* =.045, 95% CI: − 0.05 and 9.52), interest in the field of nursing (t(284)=-3.855, *p* <.001, 95% CI: -16.79 and − 4.83, and support from friends (t(284)=-2.626, *p* =.009, 95%CI: 95% CI: -13.58 and − 1.92. Nursing students reported moderate levels of self-efficacy (M = 30.22, SD = 5.62), academic burnout (M = 42.08, SD = 17.84), and quality of learning experience (M = 58.97, SD = 11.19). However, the participants reported severe psychological distress (M = 32.20, SD = 9.75). Academic burnout was negatively correlated with self-efficacy (*r*=-.514, *p* <.001), psychological distress (*r*=-.585, *p* <.001), and the quality of learning experience (*r*=-.407, *p* <.001). The weakest correlation with students’ academic burnout was the overall quality of learning experience, while the strongest correlation was psychological distress.

**Conclusion:**

Our results suggest that academic institutions should ensure realistic academic workloads and expectations that align with students’ capabilities while avoiding excessive coursework or unrealistic deadlines. Time management skills, coping strategies, problem-solving, goal setting and prioritization should be incorporated into the programs to help students develop a balanced approach to reduce academic burnout and psychological distress.

**Clinical trial number:**

Not applicable.

## Background

Academic burnout refers to a syndrome characterized by three dimensions: emotional exhaustion from intense academic pressure, depersonalization manifested in cynicism toward academic tasks (cynicism), and a diminished sense of personal accomplishment (inefficacy) [1]. Mahmoud et al. (2023) defined academic burnout as the state of exhaustion and disinterest experienced by students due to the demands and expectations associated with their studies. It reflects a sense of inadequacy and mental exhaustion in response to the stressors and lack of resources necessary to fulfill academic responsibilities [2].

This issue is particularly prevalent among nursing students, with studies indicating burnout rates ranging from 25 to 60% [3–6]. Nursing students confront unique stressors beyond the inherent demands of academic learning, including demanding curricula, extensive study plans, poor time management, and challenging clinical experiences such as witnessing patient mortality and suffering, exposure to workplace hazards, and managing complex relationships [5, 7–9]. These factors contribute significantly to high levels of stress and burnout, negatively impacting students’ emotional, physical, and mental well-being. Symptoms include fatigue, frustration, anxiety, and disengagement from academic and clinical activities, which can hinder both learning and patient care quality [5, 8, 9]. Understanding academic burnout is crucial for promoting student well-being, engagement, and future professional competence, as its symptoms can lead to reduced academic performance and chronic mental health issues [4, 5, 7, 9].

The quality of learning experience plays a significant role in burnout. Effective communication between faculty and students, along with supportive relationships and constructive feedback, can mitigate stress and enhance learning outcomes [8, 10, 11]. However, poor learning conditions can exacerbate burnout, underscoring the need to improve educational environments [2, 8]. In addition, self-efficacy, or one’s belief in their capabilities, significantly impacts motivation and learning outcomes [12–14]. Higher self-efficacy correlates with better academic performance, while low self-efficacy can lead to increased stress and burnout [12, 15–17]. Similarly, psychological distress, which encompasses symptoms of anxiety and depression, can heighten vulnerability to academic burnout, creating a cycle of disengagement and exhaustion [18–20]. Given its consequences, early identification and intervention are critical to safeguarding students’ mental health and career sustainability [1, 8].

While self-efficacy and psychological distress are well-documented correlates of burnout [12–20], the role of quality of learning experience remains understudied and its relationship with burnout lacks contemporary evidence, particularly in nursing education. Additionally, no studies have explored these dynamics among nursing students in Saudi Arabia, where cultural and institutional factors may uniquely influence burnout.

### Purpose

This study aims to examine the prevalence of academic burnout, self-efficacy, psychological distress, and the quality of learning experience among undergraduate nursing students. It will also investigate differences in academic burnout based on demographic characteristics and explore the relationships between these variables. By addressing these gaps, the study can inform targeted interventions to improve nursing education and student well-being.

## Methodology

### Research design

A cross-sectional design using a quantitative method was incorporated in this study.

### Population, sample and sampling techniques

A purposive sampling technique was used in this study. The total study population was 1108 undergraduate students who were studying in the College of Nursing. The inclusion criteria were: (1) Undergraduate nursing students enrolled in the College of Nursing from the first to the fourth year; (2) Aged between 18 years or older; and (3) Involved in the Nursing or Midwifery Program. The exclusion criteria were students in internship year, as they are not taking any courses during their internship year and considered as graduated students from the Nursing or Midwifery programs. Sample size was calculated using the z-score. A 95% confidence level and a 5% margin of error were used to calculate the sample size. The minimum number of participants required for this study was (*n* = 285) undergraduate nursing students.

### Research setting

The study was conducted in the College of Nursing at one of the academic institutions located in Saudi Arabia.

### Research instruments

The online survey included three parts as follows:

#### Demographic data

The demographic data contained 11 questions which are students’ age, level of education, undergraduate program, GPA, having interest in the nursing field, English proficiency level, marital status, place of residence, support from friends, support from family, or having financial issues.

#### Academic burnout scale

Academic Burnout was measured by Maslach-Burnout Inventory-Student Survey (MBI-SS) Schaufeli et al. (1996). The MBI-SS consisted of 15 items that were grouped into three subscales: Exhaustion (5 items), Cynicism (4 items), and Academic Efficacy (6 items). All items were scored on a 7-point frequency rating scale ranging from “0” (never) to “6” (always). The academic efficacy items (10, 11, 12, 13, 14, 15) were reversed coded. High scores on exhaustion and cynicism, and low scores on academic efficacy are indicative of academic burnout. Zis et al. (2021) [21] divided each subscale score into quartiles, with the 25th percentile or lower scoring meant low scores whereas the 75th percentile or higher scores indicated high scores. In this study, the reliability of subscales was 0.907 for exhaustion, 0.887 for cynicism, 0.863 for academic efficacy, and 0.890 for overall academic burnout scale.

### General self-efficacy scale

A General Self-Efficacy Scale was used to measure the participants’ self-efficacy. The survey was developed by Schwarzer and Jerusalem in 1979 [22]. This scale contained 10 items which evaluated the general sense of perceived self-efficacy. Participants must indicate the amount of agreement to each item, which were scored on Likert response scale from 1 (not at all true) to 4 (exactly true). The total score ranged between 10 and 40, with a higher score indicating more self-efficacy. In this study, interquartile scores were used for interpreting the results. Scores of 27 or lower indicated a low self-efficacy, scores between 28 and 33 indicated a moderate self-efficacy, and scores of 34 or higher indicated a high self-efficacy. In this study, the reliability of the scale was 0.898.

### Kessler psychological distress scale

Kessler Psychological Distress Scale (K10) was used to assess psychological distress [23]. This scale contained 10 items about emotional states. The items were measured on a Likert response scale from 1 (all the time) to 5 (none of the time). The scores were evaluated depending on the total. The total score ranged between 10 and 50, with a higher score indicating a high level of psychological distress. The reliability coefficient was reported in a previous study by Kessler, et al. (2003) for K10 scale was 0.93 [23]. The interpretation of scores was presented in previous study by Kessler et al. (2003) [23]. The reliability of the scale in this study was 0.932.

### Quality of learning experience scale

The Quality of Learning Experience scale was used to examine the quality of learning experience of nursing students. The scale was developed by Neumann [24] and it contained 15 items covering five aspects of learning experience. The subscales were recourses (2 items), content (4 items), learning flexibility (3 items), student-faculty formal and informal (4 items), and student involvement (2 items). The scores of this scale ranged from 1 (very poor) to 5 (excellent). The total score ranged between 15 to75, with a lower score indicated a poor quality of learning and a higher score indicated an excellent quality of learning. In this study, the Cronbach alpha reliability was 0.780 for resources, 0.820 for content, 0.826 for learning flexibility, 0.892 for student-faculty formal and informal contact, 0.842 for involvement, and 0.940 for overall quality of learning experience.

### Statistical analysis

The data was compiled and statistically analyzed using SPSS version 28. Descriptive statistics including frequencies and percentages were used to calculate, describe, and summarize collected research data and to answer the first research question. An independent t-test and ANOVA were used to answer the second question to test the significant difference in students’ academic burnout on their demographic characteristics. A Pearson correlation coefficient was used to answer the third research question by examining the strengths and direction of the relationships between the study variables. Cohen [25] guidelines for interpreting the strength of the correlation coefficient value was used in this study. The values of *r* =.10 to 0.29 indicated a weak correlation, *r* =.30 to 0.49 indicated a moderate correlation, and *r* =.50 to 1.0 indicated a strong correlation. A p-value of 0.05 was regarded as statistically significant for all tests.

### Ethical consideration

IRB approval was obtained in accordance with the Declaration of Helsinki from an academic institution located in Riyadh, Saudi Arabia (PNURSP2024R447). Also, informed consent to participate was obtained from all the participants in the study. The participants were informed that data privacy and confidentiality were maintained and protected. Prior to data collection, the participants received a clear description about the study including the objectives and the methodology. The potential participants had the right to participate in the study. They were informed that their participation was voluntary, and they had the right not to answer any of the questions or withdraw from the study without any penalty. The potential participants who agreed to participate signed an informed consent. Participants were informed that data will be protected and will be used for research purposes only. Contact information with the authors was provided on the first page of the questionnaire. The participants were given the chance to ask questions and ask for clarification.

### Data gathering procedures

After receiving the IRB approval, the Students’ Affairs in the College of Nursing were asked to distribute the self-administered questionnaire to all students through the academic email. A barcode of the questionnaire was distributed on the bulletin-board in the College of Nursing after getting the approval from the Students’ Affairs. The co-authors used face-to-face recruitment for data collection to encourage the potential participants for participation and engagement in our research. Microsoft Forms was used for data collection. Data was collected in two months between March and April of 2024. The participants were asked to fill-out the survey once, and it took 7 to 15 min to complete the survey.

## Results

Table 1 shows that the total number of participants who agreed to participate were 286 students. The mean age of the students was 20.39 years (SD = 2.08). Approximately half of the students were aged between 18 and 20 years, while the remaining students were 21 years or older. Around an equal number of the participants were studying in the first, second and fourth year (26.6%, 26.2%, and 27.3% respectively), while only 19.9% of them were studying in the third year. Of the participants, 75.2% were studying in the Bachelor of Nursing program, while only 24.8% were studying in the Bachelor of Midwifery program. Most of the participants (72.7%) indicated that their GPA was above 3.75, (83.9%) have interest in the field of nursing, and their English proficiency level is intermediate (67.1%). The majority were single (98.3%), resided with their parents (90.6%), had support from friends (85.3%) and family (91.6%), and had no financial issues (78%).

**Table 1:** Student’s demographics and the differences in the mean scores on levels of academic burnout (*n* = 286)

Variable	*n* (%)	Mean (SD)	t/F	*p*	95% CI	
					Lower	Upper
**Age**						
≤20	145 (50.7%)	42.26 (18.29)	0.174	0.862	-3.79	4.53
≥21	141 (49.3%)	41.89 (17.43)				
**Level of Education**						
First year	76 (26.6%)	40.93 (18.17)			36.78	45.78
Second year	75 (26.2%)	42.01 (2.07)	0.646	0.586	37.88	46.14
Third year	57 (19.9%)	40.61 (2.68)			35.25	45.98
Fourth year	78 (27.3%)	44.33 (15.55)			40.82	47.84
**Undergraduate Program**						
Nursing	215 (75.2%)	43.45 (17.44)	2.27	0.024*	0.55	10.45
Midwifery	71 (24.8%)	37.94 (18.52)				
**GPA**						
≤3.74	78 (27.3%)	45.53 (18.49)	2.011	0.045*	-0.05	9.52
≥3.75	208 (72.7%)	40.79 (17.46)				
**Interest in Nursing**						
Yes	240 (83.9%)	40.34 (17.14)	-3.855	<0.001*	-16.79	-4.83
No	46 (16.1%)	51.15 (18.84)				
**English Proficiency Level**						
Beginner	45 (15.7%)	48.69 (19.34)			42.88	54.5
Intermediate	192 (67.1%)	41.46 (16.38)	4.328	0.014*	39.13	43.8
Advanced	49 (17.1%)	38.43 (20.59)			32.52	44.34
**Marital Status**			-1.281	0.201	-26.12	5.53
Single	281 (98.3%)	41.90 (17.80)				
Married	5 (1.7%)	52.20 (19.45)				
**Residence**		24.08 (17.84)				
With Parents	259 (90.6%)	41.97 (17.49)			39.83	44.1
With relatives	10 (3.5%)	47.10 (24.37)	0.438	0.646	29.67	64.53
Alone	17 (5.9%)	40.88 (20.21)			30.5	51.27
**Support from Friends**						
Yes	244 (85.3%)	40.94 (17.71)	-2.626	0.009*	-13.58	-1.92
No	42 (14.7%)	48.69 (17.37)				
**Support from Family**						
Yes	262 (91.6%)	41.58 (17.37)	-1.583	0.115	-13.48	1.46
No	24 (8.4%)	47.58 (22.04)				
**Financial Issues**						
Yes	63 (22%)	45.38 (17.68)	1.668	0.096	-7.62	9.23
No	223 (78%)	41.15 (17.81)				

t, F: test of significance; p: Significant at the 0.05 level; SD: Standard deviation; CI: Confidence interval of the Difference

The differences between the student demographics’ mean scores on levels of academic burnout are presented in Table 1. The results showed that there is a statistically significant difference in the mean score of academic burnout levels between students who were studying in the Bachelor of Nursing program (M = 43.45, SD = 17.44) and the Bachelor of Midwifery program (M = 37.94, SD = 18.52) (t(284) = 2.270, *p* =.024), with the 95% confidence interval (CI) for the difference in mean was [0.55, 10.45]. Also, a statistically significant difference was found between students who had a GPA below 3.75 (M = 45.53, SD = 18.49) and a GPA of 3.75 or above (M = 45.53, SD = 18.49) (t(284) = 2.011, *p* =.045), with the 95% CI for the difference in mean was [-0.05, 9.52]. A statistically significant difference was also found between students who had an interest in the field of nursing (M = 40.34, SD = 17.14) and those who do not (M = 51.15, SD = 18.84) (t(284)=-3.855, *p* <.001), the 95% CI for the difference in mean was [-16.79, -4.83]. Students who have support from their friends (M = 40.94, SD = 17.71) and those who do not (M = 48.69, SD = 17.37) were compared. The results also indicated a significant difference in support from friends between the two groups, t(284)=-2.626, *p* =.009, 95% CI for the difference in mean was [-13.58, -1.92].

In addition, a one-way ANOVA was conducted to compare the effects of three different English proficiency levels on students’ academic burnout. The results indicated a statistically significant effect, F(2, 283) = 4.328, *p* =.014). Beginner (M = 48.69, SD = 19.34), intermediate (M = 41.46, SD = 16.38), and advanced levels of English proficiency (M = 38.43, SD = 20.59) were compared. Table 2 shows Post hoc comparisons using the Tukey HSD test. It revealed that students with beginner level of English proficiency had statistically significant higher academic burnout than students with intermediate level of English proficiency (*p* =.037), and than those with advanced level of English proficiency (*p* =.014). However, students with intermediate level of English proficiency had non-statistically significant higher academic burnout than students with advanced level of English proficiency (*p* =.530). There were no statistically significant differences in students’ academic burnout on their ages (*p* =.862), level of education (*p* =.586), marital status (, place of residence, support from family, and financial issues.

**Table 2:** Post hoc test- multiple comparisons between the variables

Variable			Mean Difference	*p*	Lower	Upper
Level of Education	1st year	2nd year 3rd year 4th year	-1.08 0.23 -3.40	0.983 0.986 0.640	-8.60 -7.78 -10.84	6.44 8.41 4.05
	2nd year	3rd year 4th year	1.40 -2.32	0.970 0.853	-6.72 -9.80	9.52 5.15
	3rd year	4th year	-3.72	0.631	-11.77	4.33
English Proficiency Level	Beginner	Intermediate Advanced	7.23* 10.26*	0.037 0.014	0.34 1.68	14.11 18.84
	Intermediate	Advanced	3.03	0.530	-3.62	9.69
Residence	With Parents	With relatives Alone	-5.13 1.08	0.646 0.968	-18.71 -9.46	8.44 11.63
	With relatives	Alone	6.22	0.658	-10.57	23.00

*. The mean difference is significant at the 0.05 level

Table 3 shows students reported a moderate level of self-efficacy (M = 30.22, SD = 5.62) and are likely to have severe psychological distress (M = 32.20, SD = 9.75). They also have moderate levels of exhaustion (M = 21.09, SD = 7.83), cynicism (M = 15.68, SD = 3.42), and academic efficacy (M = 25.69, SD = 7.65). They also reported a moderate level of overall academic burnout (M = 42.08, SD = 17.84). Students reported a high level of resources (M = 7.99, SD = 1.61), yet they showed moderate levels of content (M = 15.88, SD = 3.48), learning flexibility (M = 11.85, SD = 2.76), student-faculty formal and informal contact (M = 15.89, SD = 3.48), and involvement (M = 7.63, SD = 2.05). Students also reported an excellent level of overall quality of learning experience (M = 58.97, SD = 11.19).

**Table 3:** Descriptive analysis of the variables (*n* = 286)

Variable	Mean	SD	Low *n* (%)	Moderate *n* (%)	High *n* (%)
1. Self-Efficacy	30.22	5.62	76 (26.6%)	126 (44.1%)	84 (29.4%)
2. Psychological Distress	32.20	9.75	63 (22.0%)	46 (16.1%)	177 (61.9%)
3. Exhaustion	21.09	7.83	81 (28.3%)	119 (41.6%)	86 (30.1%)
4. Cynicism	15.68	3.42	95 (33.2%)	99 (34.6%)	92 (32.2%)
5. Academic Efficacy	10.31	7.65	73 (25.5%)	136 (47.6%)	77 (26.9%)
6. Overall Burnout	42.08	17.84	72 (25.2%)	143 (46.9%)	80 (28.0%)
7. Resources	7.99	1.61	93 (32.5%)	92 (32.2%)	101 (35.3%)
8. Content	15.88	3.18	81 (28.3%)	114 (39.9%)	91 (31.8%)
9. Learning Flexibility	11.58	2.76	86 (30.1%)	123 (43.0%)	77 (26.9%)
10. Contact	15.89	3.48	82 (28.7%)	123 (43.0%)	81 (28.3%)
11. Involvement	7.63	2.05	72 (25.2%)	115 (40.2%)	99 (34.6%)
12. Overall Quality of Learning	58.97	11.19	72 (25.2%)	134 (46.9%)	80 (28.0%)

Table 4 shows the relationship between study variables. There were strong negative statistically significant correlations between the overall academic burnout and self-efficacy (*r*=-.514, *p* <.001) and psychological distress (*r*=-.585, *p* <.001). That is when self-efficacy and psychological distress decreased, students’ academic burnout increased. However, there was a moderate negative correlation between the overall academic burnout and content (*r*=-.362, *p* <.001), learning flexibility (*r*=-.320, *p* <.001), student-faculty formal and informal contact (*r*=-.416, *p* <.001), involvement (*r*=-.366, *p* <.001), and overall quality of learning experience (*r*=-.407, *p* <.001). That is when content, learning flexibility, student-faculty formal and informal contact, involvement, and the overall quality of learning experience decrease, the overall academic burnout increases. Also, a weak negative statistically significant correlation was found between resources and the overall academic burnout (*r*=-.199, *p* =.001). That is, when resources decrease, the overall academic burnout increases. As shown in Table 4, the weakest correlation with the overall academic burnout is resources while the strongest correlation is psychological distress.

**Table 4:** Correlation between study variables and academic burnout subscale (*n* = 286)

Variable	Cronbach Alpha	1	2	3	4
1. Self-Efficacy	0.898	1			
2. Psychological Distress	0.932	0.426^**^	1		
3. Overall Quality of Learning Experience	0.940	0.422^**^	0.389^**^	1	
4. Overall Academic Burnout	0.890	− 0.514^**^	− 0.585^**^	− 0.407^**^	1

**Significant at the 0.01 level

## Discussion

This research study was conducted to examine the differences in the mean scores of students’ academic burnout on their demographic characteristics. The results yielded that there was no significant difference in the mean score of students’ academic burnout on their age. Our result is inconsistent with previous studies that reported high levels of burnout among younger students compared to older students [4, 9]. These differences may be due to the relatively small age differences between most of the participants.

Our results showed that there was no significant difference in the mean score of academic burnout on the levels of education. This result is inconsistent with previous study that was conducted by Rahmatpour et al. (2019), where the authors indicated that students in different academic years may experience varying levels of burnout [26]. Other researchers indicated that students at higher levels of education experienced significant levels of academic burnout [27]. This inconsistency could be due to that nursing students at different levels of education often encounter similar stressors and indeed had similar levels of burnout.

The findings of the study validated the existence of a significant difference in the mean score in the academic burnout between nursing students who were studying in the nursing program and the midwifery program. Contrary to this result, no significant difference was found in the mean score of academic burnout level between students in both programs [28]. This suggests that students in different programs have different areas of focus and coursework. Also, students encounter specific demands and challenges, which contribute to differences in burnout levels.

In this research, there was a statistically significant difference in the mean scores of the academic burnout between students with GPA lower than 3.74 and higher than 3.75. Our results are inconsistent with previous studies that were conducted by Aghajari et al. (2018) [8] and Karabulut et al. (2021) who reported that whenever the GPA increased, the level of Academic burnout also increased [29]. This suggests that high achieving students exhibit perfectionistic tendencies and set excessively high expectations and standards for themselves. These students perform at a high level and consistently feel pressure, which consequently contributes to chronic stress and academic burnout.

In this study, a statistically significant difference was also found in the levels of academic burnout between students who have interests in the field of nursing and those who have do not. A lack of interest in one’s field of study can cause academic burnout, aligning with previous research findings [26, 29, 30]. However, previous studies also reported opposite findings where no significant differences exist [5, 9, 31–33]. Our results suggest that students interested in nursing are more likely to be motivated to succeed in their academic pursuits and may see their academic studies as more relevant and meaningful. This perception of relevance and passion for nursing can help them overcome challenges and prevent burnout.

Students who reported a beginner level of English proficiency have a statistically significant different levels of academic burnout when it compared with those who reported intermediate and advanced level of English proficiency. This result is in line with previous studies that were conducted by Liu (2023) and Zuo et al. (2024), suggesting that students with language barriers may face challenges in understanding lectures, participating in class discussions, or comprehending course materials [34, 35]. Language barriers can lead to increased stress and frustration, as they have strain to keep pace with academic demands. The additional effort required to understand and communicate in a language that is not their native language can contribute to academic burnout [36].

There were no statistically significant differences in mean scores of academic burnout between students who are single and married, between students who reside with their parents, relatives or alone, and between those who have financial issues and do not which was inconsistent to previous study [26, 37]. This difference suggests that academic burnout is a complex and multifaceted issue, and various factors contribute to it.

In addition, a statistically significant difference was found in the levels of academic burnout between students who have support from their friends that those who do not. This result is consistent with previous studies [5, 6, 9] which suggest that social support from friends can play a crucial role in mitigating the negative effects of academic burnout among nursing students.

The study found that Saudi nursing students exhibited moderate level of self-efficacy (30.22 ± 5.62), exceeding findings from Mei et al. (2022) in China [37]. These differences could be due to the transition to the rigorous nursing curriculum and clinical training and lack for mastery experiences, which ultimately influence their self-efficacy.

Psychological distress was higher in Saudi students (32.20 ± 9.75) compared to the moderate level reported by Chen et al. (2023) [30]. This suggests that intense workload and demanding clinical practice, dealing with emotional situations, and role ambiguity as nursing students can be a significant source of psychological distress among nursing students [38, 39].

The results of this study showed moderate levels of emotional exhaustion, cynicism, academic efficacy, and overall academic burnout. However, previous studies reported high emotional exhaustion, cynicism, overall academic burnout, yet low academic efficacy level was reported [4, 9, 40, 41]. These differences can be attributed to variations in the demographic profiles of the participants and culture, in addition to differences in measurement tools used in each study.

Participants of this study reported an excellent level of overall quality of learning experience. Our result is consistent with the study conducted by Ekstedt et al. (2019) [42]. This suggests that students may value a variety of instructional approaches and have engaging, experienced, supportive, and accessible faculty members. Nursing students may have the opportunity to collaborate and interact with their peers in group discussions, case studies, and projects. They may also appreciate their willingness to access resources and facilities such as well-equipped simulation labs, libraries, online learning platforms, and technology.

In the present study, the relationship between academic burnout and self-efficacy was inversely related, which means that a high level of self-efficacy leads to a low level of academic burnout. This finding is consistent with the results of previous research [1, 30, 43]. These results are attributed to students with high self-efficacy prone to have confidence, adaptability, and management for academic challenges. These students are more likely to deal with academic difficulties and stressors by using effective coping strategies. Students with high self-efficacy better regulate their stress levels and prevent burnout from escalating.

In the study conducted by Chen, et al. (2023), it was revealed that academic burnout and psychological distress were positively correlated [30]. In contrast with this study’s findings which showed there is a negative correlation between these variables. The inverse relationship could be attributed to the fact that if the students were stressed out, their productivity would rise, which would lead them to show more seriousness about their studies. In addition, students may seek out social support, employ coping strategies to manage their emotions, and become more resilient in the face of stressors. These factors can act as barriers against the development of academic burnout. Also, experiencing physical or emotional distress when caring for patients and exposing them to emotionally challenging situations can contribute to burnout. Lack of support, time management, and coping mechanisms to deal with stressors, and inability to balance between the demands of nursing education with personal life could result in increased stress and burnout. Undergoing high levels of stress and pressure for prolonged periods, having challenges to focus and retain information, and perform well academically, can adversely contribute to academic burnout.

Our study depicted that there was a moderate negative correlation between the overall academic burnout and overall quality of learning experience, which was consistent with the findings reported by Aghajari et al. (2018) [8]. This suggests that students become motivated to learn and actively engage in their academic pursuits if they find their coursework interesting, relevant, and meaningful. Students are less likely to develop burnout if they have a sense of engagement and accomplishment and have access to the needed resources. In addition, the rigorous curriculum with a heavy workload, and the demanding nature of the coursework, clinical rotations, and exams, contribute to the increased academic burnout among students.

The results of this research highlight the need for implementing support systems within this academic institution where the data was collected from. Conducting workshops or training sessions that focus on enhancing self-efficacy, stress management, and language support services, can be essential in combating academic burnout. There is a need for mental health resources and interventions tailored to the stressors faced in nursing education. Supportive learning environments that prioritize student well-being and foster a sense of belonging, while addressing perfectionism should be emphasized. Recognizing that each student’s experience with burnout and self-efficacy may be unique, it is important to provide individualized support. This can involve offering counseling services, academic mentoring, or personalized academic support. Encouraging students to reflect on their strengths, set realistic goals, and develop strategies to overcome challenges can enhance their self-efficacy and reduce the risk of burnout.

### Limitations

There are a few limitations to this study. Firstly, the sample was collected from one academic institution, which may restrict the applicability of the findings to a wider population of Saudi nursing students. With a larger sample, the results would be of more statistical significance and be more relevant to a broader range of students. Secondly, relying solely on survey data can lead to biased results. Students’ self-reported experiences may not always be entirely accurate, and the survey format may have limited the collection of detailed information. Thirdly, the use of outdated findings as reference points means the current study cannot definitively determine if the observed effects are consistent with or divergent from past research. Finally, as this is the first study of these specific variables among Saudi nursing students, making comparisons to existing research is challenging. Future studies, incorporating diverse data collection methods, and referencing current research would provide a more comprehensive understanding of these factors in the Saudi nursing student population. In addition, the attrition rate of the participants was 25.8%, which could threaten the validity of the findings. Future research could consider using personalized communication and regular follow-up to enhance the potential participants’ involvement and remind them of the study and its significance. Incentives such as gift cards and small financial incentives could also be used to facilitate participation.

## Conclusion

The study found moderate levels of academic burnout among Saudi undergraduate nursing students, with significant differences based on individual and academic factors. Students with lower GPAs, beginner-level English proficiency, or a lack of interest in nursing reported higher burnout, while those with strong social support from friends experienced lower burnout levels. These findings suggest that both academic challenges and personal engagement play key roles in burnout susceptibility.

Further analysis revealed strong negative correlations between burnout and self-efficacy (*r* = −.514) as well as psychological distress (*r* = −.585), indicating that students with lower confidence in their abilities or higher emotional strain were more likely to experience burnout. Additionally, a moderate negative association was found between burnout and the quality of the learning experience (*r* = −.407), particularly in areas such as faculty interaction, course content, and learning flexibility. This suggests that a supportive and engaging academic environment may help reduce burnout.

Overall, the study underscores the complex interplay of psychological, academic, and social factors in academic burnout among nursing students. Interventions aimed at boosting self-efficacy, improving learning experiences, and strengthening peer support networks could be effective in mitigating burnout and enhancing student well-being. Future research should further explore these relationships to develop targeted strategies for at-risk student populations.

## Data Availability

The datasets generated and/or analyzed during the current study are not publicly available due to data privacy but are available from the corresponding author upon reasonable request.
